# A test of the mechanistic process behind the convergent agonistic character displacement hypothesis

**DOI:** 10.1093/beheco/arae072

**Published:** 2024-09-14

**Authors:** Shannon Buckley Luepold, Sandro Carlotti, Gilberto Pasinelli

**Affiliations:** Swiss Ornithological Institute, Seerose 1, 6204 Sempach, Switzerland; Department of Evolutionary Biology and Environmental Studies, University of Zürich, Winterthurerstrasse 190, 8057 Zürich, Switzerland; Swiss Ornithological Institute, Seerose 1, 6204 Sempach, Switzerland; Swiss Ornithological Institute, Seerose 1, 6204 Sempach, Switzerland; Department of Evolutionary Biology and Environmental Studies, University of Zürich, Winterthurerstrasse 190, 8057 Zürich, Switzerland

**Keywords:** birdsong, competitor recognition, convergent agonistic character displacement, heterospecific aggression, interspecific competition, mixed singing, territoriality

## Abstract

In this era of rapid global change, understanding the mechanisms that enable or prevent species from co-occurring has assumed new urgency. The convergent agonistic character displacement (CACD) hypothesis posits that signal similarity enables the co-occurrence of ecological competitors by promoting aggressive interactions that reduce interspecific territory overlap and hence, exploitative competition. In northwestern Switzerland, ca. 10% of *Phylloscopus sibilatrix* produce songs containing syllables that are typical of their co-occurring sister species, *Phylloscopus bonelli* (“mixed singers”). To examine whether the consequences of *P. sibilatrix* mixed singing are consistent with CACD, we combined a playback experiment and an analysis of interspecific territory overlap. Although *P. bonelli* reacted more aggressively to playback of mixed *P. sibilatrix* song than to playback of typical *P. sibilatrix* song, interspecific territory overlap was not reduced for mixed singers. Thus, the CACD hypothesis was not supported, which stresses the importance of distinguishing between interspecific aggressive interactions and their presumed spatial consequences.

## Teaser

When your neighbor is another species, does producing a similar signal help to keep your territories separate? One hypothesis suggests that it does and that this benefit could cause interspecific signal similarity to evolve. We combined an experiment and observational data to test 2 key predictions of this hypothesis in 2 songbird species. We found that song similarity did enhance competitor recognition and territorial behavior, but this behavior did not reduce territory overlap.

## Introduction

Behavioral interactions between closely related animal species affect both their evolution and ecology, with important implications for conservation and species co-occurrence (Farwell and Marzluff 2013; Weber and Strauss 2016; Tobias and Pigot 2019). An early idea that has received renewed theoretical and empirical interest is convergent agonistic character displacement (CACD), first proposed by Cody (1969). This hypothesis states that when 2 species are in exploitative competition, signals should converge to facilitate competitor recognition by the other species. The reasoning is that, if convergent heterospecifics are “misidentified” as conspecifics by a competitor species, the resulting aggressive interactions will lead to the formation of mutually exclusive territories and thus reduce the negative consequences of competition for a limited resource. The CACD hypothesis, therefore, makes 2 key predictions: (1) behavioral responses to heterospecifics with convergent signals will be more aggressive than responses to heterospecifics with species-typical signals, and (2) territories of individuals with convergent signals should overlap less with the territories of their heterospecifics relative to the territories of individuals with species-typical signals.

Grether et al. (2009) formalized Cody’s verbal arguments in a theoretical model and established convergence in territorial signals as part of the larger framework of agonistic character displacement. Since then, numerous empirical studies in diverse geographic regions have invoked CACD as the explanation for patterns of similarity in acoustic signals among co-occurring bird species (Tobias and Seddon 2009; Tobias et al. 2014; Losin et al. 2016; Souriau et al. 2018; Drury et al. 2020). Importantly, however, none of these studies has tested whether interspecific aggression observed to ensue from convergent signals effectively reduces interspecific territory overlap. That is, prediction 2 of the CACD hypothesis has been treated as a logical assumption rather than a testable prediction.

**Fig. 1.**
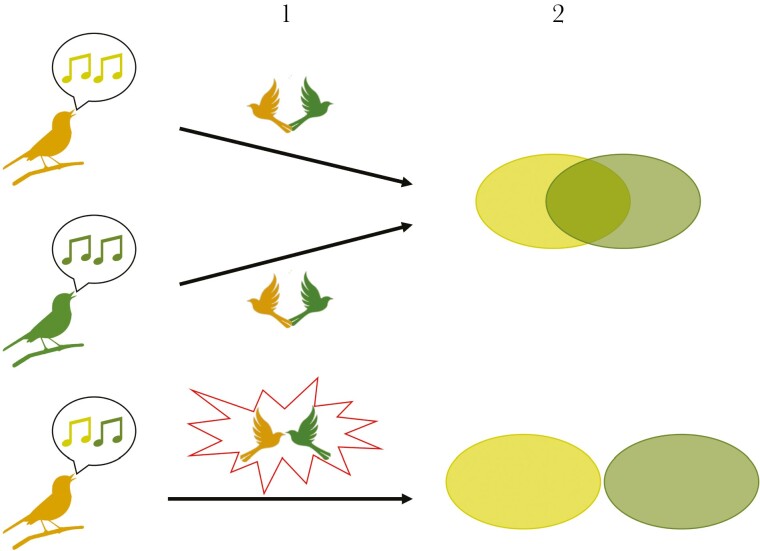
Graphical representation of the convergent agonistic character displacement (CACD) hypothesis. The lefthand column of singing birds represents the 3 song stimuli: *Phylloscopus sibilatrix* with species-typical song (top); *P. bonelli* with species-typical song (middle); *P. sibilatrix* with mixed song (bottom). The column labeled “1” represents the first set of predictions, related to heterospecific responses to the different song stimuli. The symbol of 2 birds turned away from each other indicates that the species-typical song of the other species does not elicit aggressive behavior. The red starburst indicates that *P. bonelli* responds aggressively to *P. sibilatrix* with mixed song. The column labeled “2” represents the second set of predictions and shows the level of heterospecific territory overlap that is expected to result from the behavioral interactions (or lack thereof) depicted in column 1.

**Fig. 2.**
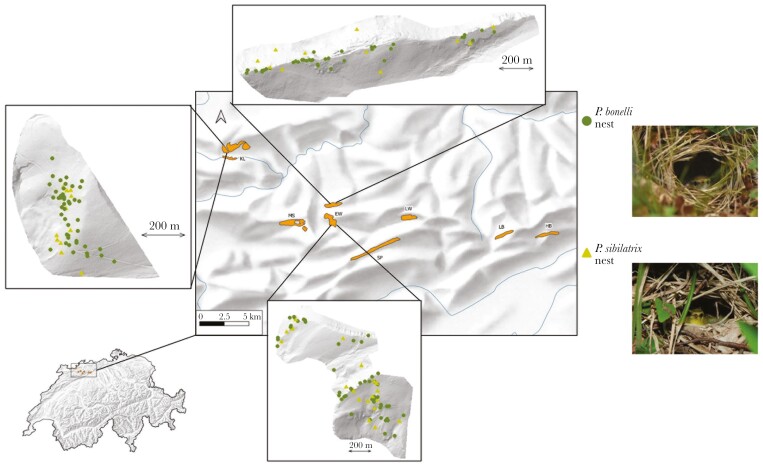
Study sites in Switzerland, zoomed in at 3 sites to illustrate nest locations of *P. bonelli* and *P. sibilatrix* within sites (including all nests 2017–2020). Basemap © swisstopo, Bern, Switzerland. Photo credits: Sandro Carlotti (*P. bonelli*) and Michael Gerber (*P. sibilatrix*).

In this study, we combined a playback experiment with observational data on territory overlap in 2 species of leaf warbler to test both predictions of the CACD hypothesis (Fig. 1). *Phylloscopus sibilatrix* and *P. bonelli* are sister species that diverged from each other ca. 4 million years ago (Alström et al. 2018). They co-occur during their breeding season in parts of central Europe (Bezzel 1977; Cramp 1992), and the area of sympatry appears to be extending due to the northward expansion and increased abundance of *P. bonelli* in Europe (Keller et al. 2020). Given their sister-species status, it is unsurprising that the ecological niches of *P. bonelli* and *P. sibilatrix* apparently overlap in various dimensions. Data from our Swiss study forests (Fig. 2) indicate they often prefer the same areas within a forest for building their ground nests, suggesting that they may compete for space in patches of good nesting habitat. Furthermore, the high similarities in morphology and dietary niches of *P. bonelli* and *P. sibilatrix* (both insectivorous species, foraging with similar tactics in the tree canopy, Glutz von Blotzheim and Bauer 1991) may result in competition for food during the breeding season, when food demand is very high. Finally, anecdotal evidence from our own fieldwork and natural history accounts (Schneider 1969; Cramp 1992; Riedinger 1995) indicates that aggression between *P. sibilatrix* and *P. bonelli* occurs in the form of chases and even physical fights (either species can be the aggressor). Thus, it seems plausible that producing a song that resembles the song of *P. bonelli* could enhance the ability of *P. sibilatrix* to establish and maintain territories among *P. bonelli*, which on average, occur at densities 11 times higher than *P. sibilatrix* (Table S1).

In the Swiss Jura Mountains, roughly 10% (*n* = 103) of *P. sibilatrix* sing songs that incorporate syllables resembling those of *P. bonelli*, a behavior often referred to as “mixed singing” (Helb et al. 1985; Dalziell et al. 2015). Although *P. bonelli* and *P. sibilatrix* occasionally hybridize (Dietzen et al. 2007), mixed-singing *P. sibilatrix* do not show evidence of mixed ancestry (Shannon Buckley Luepold, unpublished data), indicating that mixed song is the result of learning. Some have argued that copying sounds from heterospecifics (i.e. learning) confounds studies of character convergence and adaptive mimicry (Tobias and Seddon 2009; Dalziell et al. 2015). Nevertheless, as noted by Grether et al. (2009), the fact that a trait is learned does not preclude selection for convergence because learning capabilities are themselves subject to genetic influence (Wheatcroft and Qvarnström 2015, 2017; Wang et al. 2019). Furthermore, if similarity to competitor signals functions in the way the CACD hypothesis suggests it does (i.e. reduced territory overlap via enhanced competitor recognition), then this effect should be observable regardless of how similarity originated. To our knowledge, this is the first study to empirically test the entire mechanistic process posited by the CACD hypothesis—namely, that (1) signal similarity enhances competitor recognition, and (2) that the increased aggression resulting from enhanced competitor recognition reduces interspecific territory overlap. Testing prediction 2 is conditional on prediction 1 being supported.

## Methods

### Study species and song descriptions

*Phylloscopus bonelli* and *P. sibilatrix* are small, migratory passerines (~8 and 9 g, respectively, see Table S2 for detailed biometrics) that over-winter in sub-Saharan Africa. The breeding range of *P. sibilatrix* extends across much of Europe into Russia, whereas *P. bonelli* is restricted to more mountainous areas throughout the Mediterranean, western and central Europe (Cramp 1992). In our study sites, males of both species begin arriving in mid-April and sing intensively to attract females (females are not known to sing). Singing activity strongly declines after pairing (Cramp 1992; Carlotti 2020). In our study region, *P. sibilatrix* moves territories (defined as areas in which males sing and attempt to exclude other males) both between and within breeding seasons (Luepold et al. 2024b), whereas *P. bonelli* is more philopatric (Shannon Buckley Luepold and Sandro Carlotti, unpublished data). Thus, in contrast to *P. bonelli*, *P. sibilatrix* continues to establish territories in new areas throughout the breeding season, often in sites with dense populations of *P. bonelli*. Both species have mostly non-overlapping intraspecific territories, with *P. sibilatrix* territories (mean ± SE = 0.457 ± 0.004 ha, *n* = 149) being slightly larger than *P. bonelli* territories (mean ± SE = 0.318 ± 0.000 ha, *n* = 696). Both species nest on the ground and are frequently subject to high rates of nest predation (Wesołowski et al. 2009; Roncalli et al. 2016; Grendelmeier et al. 2018).

**Fig. 3.**
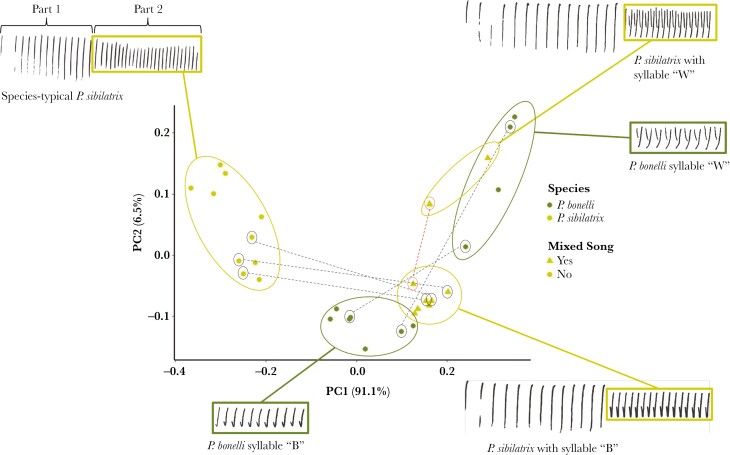
Example spectrograms and illustration of acoustic similarity between syllables in part 2 of species-typical *P. sibilatrix* song and *P. sibilatrix* mixed song, and *P. bonelli* song. Acoustic similarity was analyzed using the dynamic time-warping algorithm in the software program Luscinia (see Supporting Information for details). Each point in the plot represents an individual. Circled points in the plot connected by dashed lines indicate 2 different syllable types sung by the same individual. Spectrograms with time and frequency axes are included in the Supporting Information (Figs. S1 and S2).

The trill song of *P. sibilatrix* consists of 2 parts—an “introductory phrase” of high-frequency, staccato syllables (often given in flight) followed by a series of more rapid, lower-frequency syllables (Fig. 3). *Phylloscopus bonelli* has a diversity of syllable types (ca. 25), with any 1 individual singing between 2 and 6 syllable types (S.B.L. and S.C., unpublished data). Unlike *P. sibilatrix* trill songs, *P. bonelli* songs do not have 2 stereotypic parts (Fig. 3), but males sometimes switch syllable types within a trill (Cramp 1992; Carlotti 2020). We define mixed-singing *P. sibilatrix* (hereafter, “mixed singers”) as individuals that sing trills in which the second part is comprised of a *P. bonelli* syllable type (Fig. 3, hereafter “mixed trills”). Most mixed singers in our study region (73%, *n* = 11) alternated between singing species-typical trills and mixed trills. For 7 such individuals for which we obtained quality recordings, the mean ± SE proportion of mixed trills out of total trills was 38.9 ± 4%, *n* = 475 trills. We also recorded 1 individual that always sang mixed trills with a single type of *bonelli* syllable, 1 individual that alternated between mixed trills containing 2 different types of *bonelli* syllables, and 1 individual that invariably appended a few *bonelli* syllables at the end of an otherwise species-typical trill. Although the resemblance between *bonelli*-type syllables sung by mixed singers and syllables sung by *P. bonelli* is audible to humans and visually apparent in spectrograms, we confirmed that syllables were similar in acoustic structure by dynamic time-warping (DTW) analysis in Luscinia (Lachlan 2007) (Fig. 3, see Supporting Information for methodological details of DTW analysis).

### Study sites

Fieldwork was conducted from April to July 2017–2019 in several forests located in the Swiss Jura Mountains (Fig. 2). In most study forests, European beech (*Fagus sylvatica*) was the predominant species, with varying degrees of admixture with Scotch pine (*Pinus sylvestris*), spruce (*Picea abies*), fir (*Abies alba*), and oak (*Quercus* spp.). One forest was an admixture of oaks, Scotch pine, European hornbeam (*Carpinus betulus*), and hazel (*Corylus avellana*). Some study forests encompassed multiple hillsides which, for practical purposes, were treated as separate “subsites” (see Table S1). Study forests ranged in elevation from ca. 450 m to 1,000 m a. s. l., and in size from ca. 30 to 150 ha.

### General fieldwork and territory mapping

Mapping of *P. bonelli* and *P. sibilatrix* territories was done during 3 breeding seasons (2017–2019). Each year, we recorded the territory and nest locations of both species, beginning in mid-April with the arrival of the first males and continuing until early July (when singing activity in both species declines).

We used 2 approaches for collecting data on territory locations. For *P. bonelli*, in which the majority of individuals were unmarked, we used standard territory mapping (Bibby et al. 2000). Territory mapping of songbirds consists of visiting a site multiple times per season and marking the locations of singing individuals (as well as observations of other behaviors indicative of occupying a breeding territory, e.g. carrying nest material or food). Due to logistical constraints, survey effort varied between sites and years, but territory mapping for *P. bonelli* was annually based on 3 to 10 surveys per site per season (see Table S1). Based on individuals banded for another project in our study sites (Carlotti 2020), *P. bonelli* appears to be faithful to their initially selected territory throughout the entire breeding season. We hence assumed that birds found in the same locations during different surveys were the same individuals. For *P. sibilatrix*, the vast majority of which were marked with an aluminum ring and a unique combination of plastic color rings (2.1–2.3 mm), we used the Qfield app (OPENGIS.ch GmbH, Laax, Switzerland) on Samsung tablets to record the locations of marked males. In 2017, on each day we observed a given male, we marked his location every 3 minutes for a period of 2 hours, noting if he was singing or not for each location point. After realizing that most males focused their singing activity within the same small area (ca. 50 m radius) in a given observation period, we reduced the time spent per male per observation period to 15 min in 2018 and 2019. This allowed us to observe more males on a given day. We also searched for nests of both species, but given the time required to find nests and the aforementioned abundance of *P. bonelli*, we found only a small fraction of the *P. bonelli* nests in most sites.

### Quantifying heterospecific competitor recognition and aggression

Following numerous previous studies (Qvarnström et al. 2006; Uy et al. 2009; Freeman and Montgomery 2017; Weir and Price 2019), we considered behavioral responses to song playback as assays of both competitor recognition and aggression (assuming perceived competitors elicit a more aggressive response). We conducted a playback experiment in 2019 using recordings of local *P. sibilatrix* and *P. bonelli* males (recorded between 2010 and 2018). We created a total of 37 experimental sound files, representing the following 3 song stimuli: *P. bonelli* typical song (*n* = 15), *P. sibilatrix* typical song (*n* = 15), and *P. sibilatrix* mixed song (*n* = 7). For each experimental sound file, we used recordings of 1 individual. We also created control stimuli consisting of common wood pigeon *Columba palumbus* vocalizations (*n* = 8) to ensure that the potential responses of our study species were not due to unspecific noise emitted by speakers. The wood pigeon is a common species in our study forests and does not compete for breeding sites or food. However, after observing a complete lack of response to the control stimulus in 2 *P. sibilatrix* and 3 *P. bonelli*, we omitted the control trials to maximize the number of males we could test in the short time window before territorial aggression wanes. The date of the earliest experimental trial was 30 April, the latest was 23 May.

To control for possible effects of individual differences in song rate within a species, we standardized experimental sound files so that each *P. bonelli* stimulus had 7 trills/minute (determined based on recordings of 11 local males, recorded 2015–2017: mean = 7.08 trills/minute, range = 3.3–11 trills/minute) and each typical and mixed *P. sibilatrix* stimulus had 5 trills/minute (determined based on recordings of 22 local males, recorded in 2017: mean = 4.67 trills/minute, range = 2.6–6.5 trills/minute). As mentioned above, male *P. bonelli* have repertoires of 2 to 6 syllable types. When creating experimental files, we included at least 1 exemplar of each syllable type sung by a given individual.

From each recording, we selected several trills (7 for *P. bonelli*, 5 for *P. sibilatrix*) of good acoustic quality (i.e. with limited background noise) and used Audacity (Audacity Team 2018) to combine them into a one-minute sequence that was repeated 5 times for a 5-minute experimental sound file (.wav format). As mentioned previously, most mixed-singing *P. sibilatrix* alternate between singing species-typical trills and mixed-song trills. Because we were specifically interested in responses to the mixed trills, however, we selected only this type of trill for the creation of experimental sound files for the mixed *P. sibilatrix* stimulus. Alternating between species-typical trills and mixed-species trills in the same experimental files when testing for the effects of mixed-species trills would not have allowed distinguishing whether the responses of the tested individuals were due to the species-typical trills or the mixed trills. One of the mixed singers alternated between singing 2 types of *bonelli*-like syllables, and we included exemplars of each type in the file.

We tested the behavioral responses to the different song stimuli in 15 male *P. sibilatrix* and 15 male *P. bonelli*. We excluded some trials from analysis due to a complete lack of response to any playback or poor visibility conditions, which resulted in a total of 13 *P. sibilatrix* and 10 *P. bonelli*. The relative infrequency of mixed singers prevented us from analyzing their responses separately from those of *P. sibilatrix* with species-typical song. One of the 13 *P. sibilatrix* tested was, however, a mixed singer. His responses toward the 3 playbacks were similar to typical-singing *P. sibilatrix*, so we included them in the analysis.

Both species sing throughout the day, and trials were conducted between 0600 and 1700. For a given focal individual, we presented all song stimuli on the same day, with at least 1 h between stimuli. Each male was randomly assigned 1 of the 15 species-typical song stimuli (i.e. experimental sound files) of *P. sibilatrix* and *P. bonelli* so that each song stimulus of the species-typical song was used only once per species. The 7 song stimuli with the mixed songs were also randomly assigned to each male, such that 6 stimuli were used twice per species and 1 song stimulus was used 3 times per species. The minimum distance between birds tested on the same day was 140 m, and no neighbors were tested on the same day. The order in which song stimuli were presented was randomized between individuals. Because familiarity can influence the aggressive responses of birds to potential territorial rivals (Stoddard 1996), all tests were done using recordings from birds taken from a different study site than the focal individual. No birds were tested on the same day that they were captured, and with the exception of 3 *P. sibilatrix*, all focal birds (*P. bonelli and P. sibilatrix*) were marked with color rings for individual identification prior to testing. Excluding the *P. sibilatrix* individuals that were not captured prior to the experiment did not qualitatively change the results, so they were retained in the analysis. Males were captured with playbacks (broadcasting songs not used in the experiments) and mist nets (see Luepold et al. 2024b). In both *P. sibilatrix* and *P. bonelli*, male singing activity declines markedly after attracting a female (at least in the vicinity of the female/nest; Shannon Buckley Luepold., personal observation), and males are often seen following the female while she searches for potential nest sites or brings nesting material (Shannon Buckley Luepold, personal observation). In *P. sibilatrix*, defense of the area around the nest decreases when their mates are no longer fertile. We, therefore, only tested males when they were unpaired (determined based on singing activity and a lack of observations with females) or when their mate was still fertile (i.e. pre-incubation). The nests of all tested males were located and monitored, allowing us to determine female fertile periods.

For each experimental trial, we placed a small bluetooth speaker (JBL Go 2, Harman International, CT, USA) on a 1.5 m high wooden platform near (within 20 m) where the focal male was singing. We laid a 2-m pole next to the speaker to facilitate distance estimation. If we did not hear or see the focal bird when the trial was set to begin, we waited for 5 min to see if he would appear. If he did not, we proceeded with the trial assuming the playback would draw him in (which it usually did). We recorded male behaviors during the 5-min playback period plus 5 min post-playback. An observer (Shannon Buckley Luepold) sat in a concealed location 15–20 m from the speaker and continuously recorded the focal male’s behavior into a dictaphone. Due to the difficulty of maintaining constant visual contact with focal individuals, we recorded behaviors that we could reliably and consistently estimate from our observations: minimum distance to the speaker and number of flyovers. Both of these metrics have been used in previous studies as proxies for competitor recognition and aggression (Qvarnström et al. 2006; Tobias and Seddon 2009; Weir and Price 2019). We defined a flyover as a flight over the speaker at any vertical distance. Due to the high density of *P. bonelli* in our study forests (see Table S1), testing focal birds in acoustic isolation from neighboring individuals was impossible, and playbacks regularly elicited responses from neighbors. Accordingly, we accounted for the potential influence of neighbor interference on the responses of focal birds in our statistical analyses (see below).

We analyzed the number of flyovers and minimum distance to speaker in relation to playback type (typical heterospecific, typical conspecific, and mixed-singing *P. sibilatrix*) using Bayesian generalized linear mixed models implemented in R (R Core Team 2022) with the package *brms* (Bürkner 2017). We used the following (default) prior settings: flat priors for fixed effects, student *t* distributions for intercept and random effects. The names and descriptions of all variables included as fixed effects in both models appear in Table S3 (“Time period,” “Test order,” “Female nearby,” and “Neighbor interference” were included to account for their potential to influence observed responses and confound interpretation of treatment effects). Because each male was tested multiple times, we also included male ID as a random factor in the models. We included an interaction effect between the variables “Species” and “Playback type” to see if the 2 species differed in their responses to the different song stimuli. We also included an interaction effect between “Species” and “Test order” to account for possible differences in how the species responded to multiple playbacks. For each estimated parameter, we calculated the mean of the posterior distribution (β) and quantile-based 95% credible interval (CrI). A parameter was considered “significant” if the 95% CrI did not overlap zero, corresponding to a posterior probability (PP) ≥ 97.5% (Korner-Nievergelt et al. 2015). We also calculated the PP that *P. bonelli* responded more strongly (i.e. more flyovers or closer approach to speaker) to the mixed song stimulus relative to the species-typical heterospecific stimulus. For brevity, we report the parameter estimates for “Playback type” and “Species” in the main text and present the parameter estimates for the complete set of predictors (including potential confound variables) in the Supporting Information (see Fig. S4).

### Quantifying territorial overlap

Due to the aforementioned differences in how bird location data were collected in the field for *P. bonelli* and *P. sibilatrix*, we used different methods for delineating territories of the 2 species. For *P. bonelli*, we scanned survey maps indicating point observations of countersinging males, pairs, and so on, at each site/year. We digitized territory polygons based on these point observations using the TerriMap online tool from the Swiss Ornithological Institute (www.vogelwarte.ch/en/projects/monitoring-of-common-breeding-birds/). To delineate *P. sibilatrix* territories, we used QGIS (QGIS Development Team 2018) to digitize 1 or more territory polygons around all the singing location points for each color-ringed individual. Clusters of singing location points for the same individual were considered to be separate territories if they were separated by ≥ 150 m, or if the space between them was occupied by a conspecific male (Temrin 1984; Temrin and Jakobsson 1988). We digitized polygons at the 1: 2,500 scale.

After digitizing territory polygons, we used the overlap analysis tool in QGIS to calculate the percent overlap with *P. bonelli* territories for each *P. sibilatrix* territory. For individuals with multiple territories, we averaged the overlap values for the different territories to obtain a single value per individual. We then tested whether the mean territory overlap values of *P. sibilatrix* mixed singers differed from those of *P. sibilatrix* with species-typical songs using Bayes factor (BF) analysis in the R package BayesFactor (richarddmorey.github.io/BayesFactor/). Following (Jeffreys 1961; Kass and Raftery 1995), we consider a BF > 1 as some degree of evidence that the 2 means are different (see Table S4 for degree of evidence thresholds) and a BF < 1 as some degree of evidence that the 2 means are not different.

## Results

### Playback experiment

**Fig. 4.**
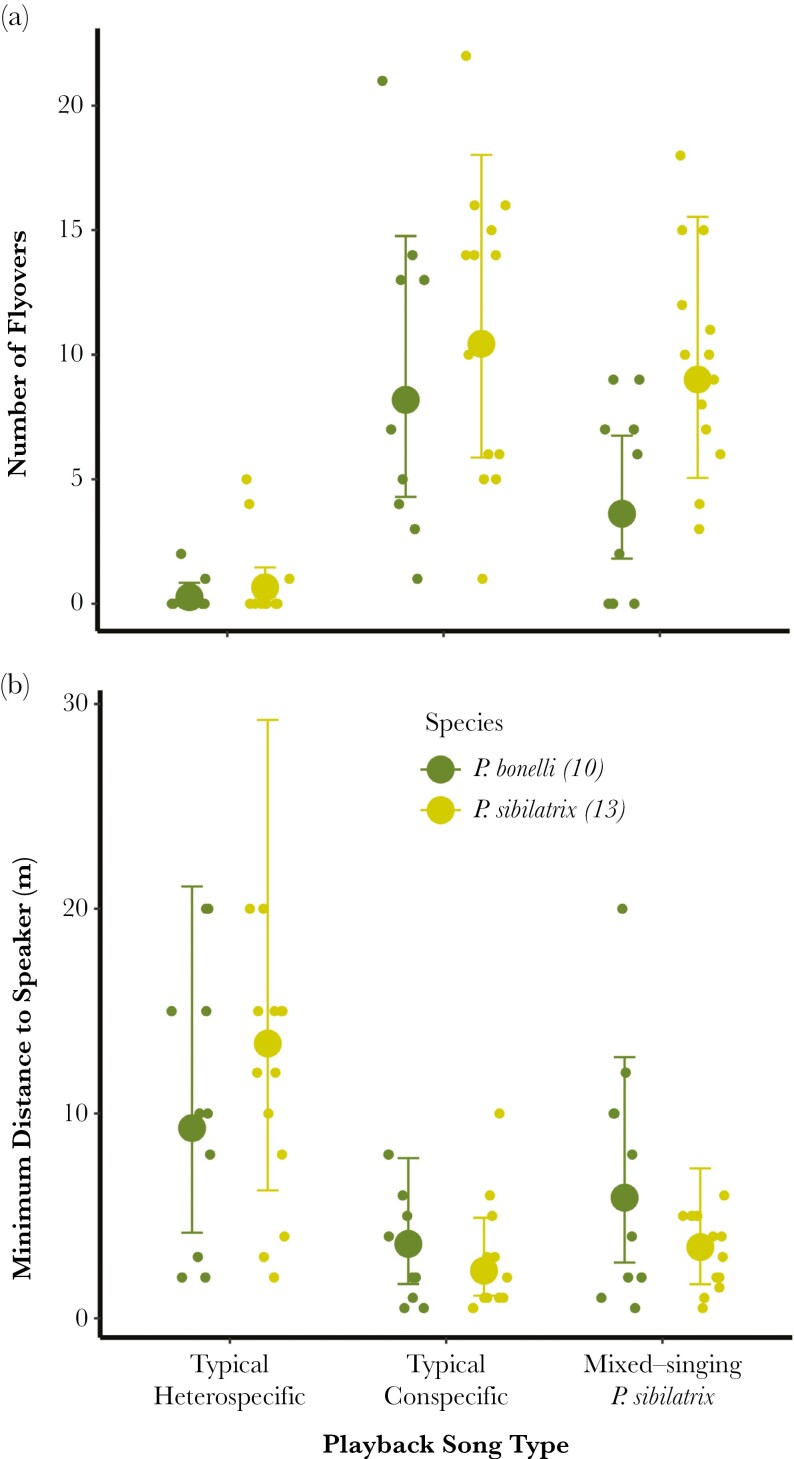
Number of flyovers (A) and minimum distance to the speaker (B) by male *P. sibilatrix* and *P. bonelli* in relation to song playback type. Large circles indicate model predictions (mean of the predictive posterior distribution), error bars represent uncertainty intervals (i.e. range of values with 2.5% to 97.5% probability) and dots are raw data points. Raw data points are jittered to reduce point overlap.

For both responses measured (minimum distance to speaker and number of flyovers), both species reacted more strongly to conspecifics than to heterospecifics (Figs. 4A and 4B). The species did not appear to strongly differ in their response to heterospecifics with species-typical song (flyover response: β_Species_sibilatrix_ = 1.13, 95% CrI: –0.33 to 2.79; distance response: β_Species_sibilatrix_ = 0.25, 95% CrI: –0.72 to 1.24). However, *P. bonelli* exhibited a stronger reaction to mixed-singing *P. sibilatrix* than to *P. sibilatrix* with species-typical song. When the response metric was a number of flyovers, there was a high certainty that the reaction of *P. bonelli* to mixed song was stronger than to species-typical heterospecific song (β_Mixed Song_ = 2.74, 95% CrI: 2.17–3.38 PP = 99.99%). Although *P. bonelli* appeared to get closer to the speaker in response to a mixed song than to species-typical heterospecific song, there was more uncertainty around this difference (β_Mixed Song_ = –0.56, 95% CrI: –1.20 to 0.09, PP = 95.46%).

### Interspecific territory overlap

**Fig. 5.**
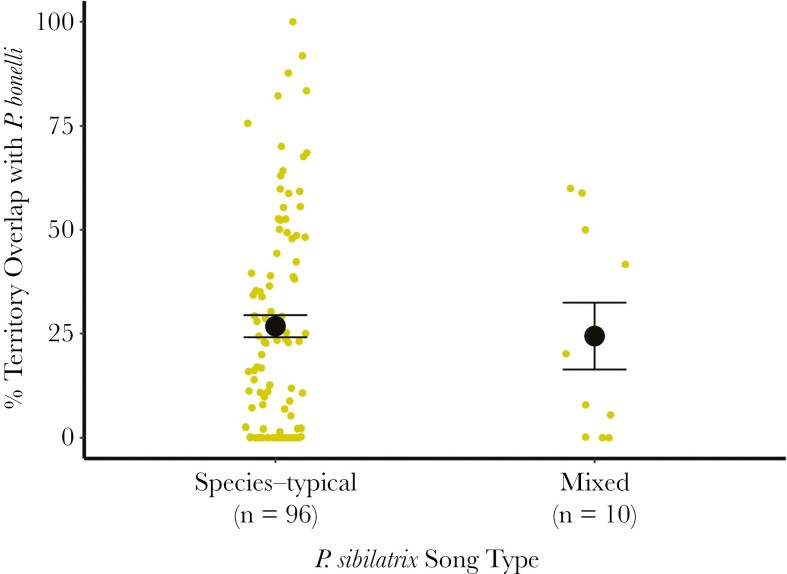
Percent overlap with *P. bonelli* territories for *P. sibilatrix* with species-typical and mixed songs. Points represent territory overlap values for individual *P. sibilatrix* males, large circles and error bars indicate mean and standard error, respectively, by song type. Raw data points are jittered to reduce point overlap.

We calculated the percent overlap with *P. bonelli* territories of the territories of 106 individually marked *P. sibilatrix*. Territory overlap between the 2 species was often substantial (i.e. ≥ 50%, Fig. 5). There was no evidence that the mean overlap with *P. bonelli* territories differed between *P. sibilatrix* with mixed song and species-typical song (BF = 0.33). For mixed singers, the mean percent overlap was 24.39% (*n* = 10, range: 0% to 59.96%), whereass for *P. sibilatrix* with species-typical song, mean percent overlap was 26.77% (*n* = 96, range: 0% to 100%).

## Discussion

In this study, we tested the following predictions of the CACD hypothesis: (1) signal convergence enhances competitor recognition by heterospecifics and (2) signal convergence reduces territory overlap with heterospecific competitors. Our playback experiment showed that *P. sibilatrix* that incorporated *P. bonelli* song elements into their songs (“mixed singers”) were more likely to be recognized as competitors by *P. bonelli* than *P. sibilatrix* with species-typical songs. However, the results of our territory overlap analysis indicated that aggressive interactions following this recognition did not reduce interspecific territory overlap. Our results are, therefore, not consistent with the process outlined by the CACD hypothesis. Below we discuss 2 non-mutually exclusive explanations for these results.

First, delineating territories and quantifying territory overlap are not trivial tasks, and results can be highly dependent on the spatiotemporal resolution of the data and on the analytical method used (Fieberg and Kochanny 2005; Anich et al. 2009; Cooper et al. 2014). Our approach here was relatively basic in that it involved circumscribing polygons around singing locations of marked and unmarked birds and then calculating 2-dimensional polygon overlap. It is possible that with field data of higher spatiotemporal resolution and a different method of territory delineation (e.g. kernel density estimation), territory delineation and overlap measurements would have been more precise. We might then have reached a different conclusion regarding interspecific territory overlap of *P. sibilatrix* individuals with mixed versus species-typical song.

Second, the current theory on CACD makes the common and intuitive assumption that winners of a contest gain access to a territory and losers leave permanently (“winner takes all”), resulting in mutually exclusive space use. Empirical studies of CACD have readily adopted this assumption, despite the fact that the form of aggressive contests and their spatial consequences can be highly variable in nature (Murray 1971; Stamps and Krishnan 1995, 1997). Field data on territory establishment in divisible space (Murray 1971; Ezaki 1995; Stamps and Krishnan 1995) as well as mechanistic models of territory formation (Stamps and Krishnan 2001; Morrell and Kokko 2005; Potts and Lewis 2014) demonstrate that more aggressive individuals (apparent “winners”) often lose ground to less aggressive individuals (apparent “losers”). Whether winning an aggressive contest per se effectively excludes an opponent from a given space often seems to depend on an opponent’s willingness to return despite being chased off or attacked. This renders the long-term spatial consequences of aggressive interactions contingent rather than determinate, which may at least partially explain why interspecific territory overlap for mixed singers remained high despite the aggressive response of *P. bonelli* to mixed songs.

The CACD hypothesis is predicated on species being in exploitative competition with one another (Grether et al. 2009, 2013), and it is possible that *P. sibilatrix* and *P. bonelli* are not true competitors for ecological resources (differences in bill morphology between *P. bonelli* and *P. sibilatrix* indicate some divergence in foraging niches, see Fig. S4). Determining how fitness of 1 species is impacted by the presence of the other would require removal experiments, which were beyond the scope of this study. Nevertheless, if aggressive responses to convergent signals function the way the CACD hypothesis posits that they do, then reduced territory overlap should be observed regardless of whether the species concerned are true competitors or not. That is, from a mechanistic perspective, behavioral responses to heterospecifics as though they were conspecifics should modify territory overlap the same way whether or not there are fitness benefits to these modifications. Our results, therefore, have important implications for the CACD and the purported benefits of signal similarity regardless of whether *P. bonelli* and *P. sibilatrix* are in fact competing for ecological resources.

The importance of distinguishing aggressive behaviors from their ecological consequences (e.g. mutually exclusive space use) was emphasized by Peiman and Robinson (2010) in their review of the ecology and evolution of heterospecific aggression. Nevertheless, it is common for studies of CACD in birds to infer that signal convergence facilitates co-occurrence via spatial partitioning based solely on a demonstration of heightened aggressive responses to playback of similar signals (Tobias and Seddon 2009; Laiolo 2012; Souriau et al. 2018). Similarly, defining “interspecific territoriality” as synonymous with aggressive behavior toward heterospecifics remains prevalent, often with the implicit assumption that mutually exclusive space use is the adaptive, functional consequence of these behavioral interactions (Losin et al. 2016; Cowen et al. 2020; Drury et al. 2020; Nesbit et al. 2023).

In this context, we believe it is worth highlighting that the very same mechanism—namely, aggression toward heterospecifics—is invoked to explain opposite biogeographical patterns in species distributions. While the above studies of interspecific territoriality and CACD argue that aggressive interactions facilitate co-occurrence, seemingly just as many argue that aggressive interactions explain species range limits and are the reason species *fail* to co-occur (Pearson and Rohwer 2000; Jankowski et al. 2010; Weir and Price 2019; Freeman et al. 2022). The fact that studies in the latter category focus primarily on cases where aggression is asymmetric (i.e. where 1 species is consistently the aggressor) cannot clarify the situation because (1) signal convergence and/or aggressive responses elicited by convergent signals are also often asymmetric (Tobias and Seddon 2009; Souriau et al. 2018; Kirschel et al. 2019) and (2) asymmetries in aggressive interactions between closely related bird species appear to be the rule (Martin et al. 2017). Boyce and Martin (2019) documented asymmetric aggression between 2 parapatric bulbul species (*Pycnonotidae*), a complete lack of aggression between 2 parapatric white-eye species (*Zosteropidae*) and asymmetric aggression between 2 sympatric flycatcher species (*Muscicapidae*). As there is no categorical difference between asymmetric aggression in co-occurring species and in parapatric species at range boundaries, Boyce and Martin (2019) argued that a demonstration of interspecific competitor recognition and aggression cannot be reliably linked to a particular pattern in species spatial distributions.

Although it is possible that (asymmetric) aggressive interactions could have opposing effects on the ability of species to coexist at a particular spatial scale, the task then becomes understanding the contexts in which these different effects occur. In a recent theoretical study, Grether and Okamoto (2022) explored whether a superior interference competitor could evolve to coexist with a superior exploitative competitor via agonistic character displacement. The outcome depended on various factors, including the strength of exploitative competition (i.e. the costs of territory overlap) and differences in fighting ability. However, territoriality was not modeled in a spatially explicit manner, and aggressive interactions always had clear “winners” and “losers.” We believe the biological realism of models of CACD could be enhanced by considering that most territories in nature emerge out of divisible space. As discussed above, rather than being discrete units that are “won” or “lost” in their entirety, territory boundaries are “negotiated” via multiple behavioral interactions between individuals (Stamps and Krishnan 1995, 1997). When individual A wins a single fight against individual B, in most cases this is unlikely to grant individual A an entire territory and leave individual B with nothing (as assumed in current models of CACD dynamics, e.g. Grether et al. 2009, Grether and Okamoto 2022). Relatedly, individuals that “lose” fights can still end up “winning” the contested space. Persisting until an aggressor gives up has been observed to be a successful strategy for gaining territory space in field studies of lizards and birds (Stamps and Krishnan 1995 and references therein).

We acknowledge that our sample size of mixed singers is small, and therefore, the results of our study are by no means conclusive with respect to the relationship between signal similarity, interspecific aggression, and territory overlap. Nevertheless, to the best of our knowledge, it is the only study that has tested whether increased similarity to the signal of a heterospecific competitor effectively reduces territory overlap with that competitor. We, therefore, encourage more empirical studies to test this assumption, upon which the CACD hypothesis critically depends.

Given the apparent cost of mixed singing in terms of aggressive interactions with *P. bonelli* and the lack of the benefit of mutually exclusive territories, non-adaptive explanations for the origin of mixed singing in *P. sibilatrix* remain fully plausible. Indeed, mixed singing may simply be an incidental consequence of song learning under certain circumstances (e.g. lack of conspecific tutors). Regardless, however, our finding that territorial aggression did not directly translate into reduced interspecific territory overlaps has important implications, particularly given recent suggestions that this mechanism has facilitated the expansion of syntopy among North American birds (Nesbit et al. 2023).

Given the rapid changes in the abundance and distribution of species occurring in the Anthropocene (Chen et al. 2011; Young et al. 2016), understanding the functional consequences of interspecific social interactions is imperative (Grether et al. 2017). Indeed, the results of this study and others indicate that increased acoustic similarity to heterospecifics often occurs in species that are locally rare or declining (Helb et al. 1985; Haavie et al. 2004; Souriau et al. 2018; Paxton et al. 2019), suggesting that this phenomenon is likely to increase in the coming decades. Our study represents the first attempt to test proposed causal links between signal convergence, behavioral interactions, and interspecific territory overlap, and we showed that aggressive interactions were apparently not linked to reduced overlap. These results highlight the importance of distinguishing between behavioral interactions and their presumed spatial consequences, and we encourage future studies addressing CACD to consider this distinction more explicitly. Only then can the mechanistic process proposed by the CACD hypothesis be empirically validated.

## Supplementary Material

arae072_suppl_Supplementary_Materials

## Data Availability

Analyses reported in this article can be reproduced using the data provided by [dataset] Luepold et al. (2024a).
