# Supplementary material for: A test of the mechanistic process behind the convergent agonistic character displacement hypothesis
Source: Behav Ecol. 2024 Sep 14;35(6):arae072. doi: 10.1093/beheco/arae072 (PMC11457480; doi:10.1093/beheco/arae072)
Supplement: arae072_suppl_Supplementary_Materials [file arae072_suppl_supplementary_materials.docx]

**Supporting Information**

A test of the mechanistic process behind the convergent agonistic character displacement hypothesis

Shannon Buckley Luepold^1, 2^, Sandro Carlotti^1^ and Gilberto Pasinelli^1, 2*^

^1^ Swiss Ornithological Institute, Seerose 1, 6204 Sempach, Switzerland

^2^ Department of Evolutionary Biology and Environmental Studies, University of Zürich, Winterthurerstrasse 190, 8057 Zürich, Switzerland

*Corresponding author. Email: gilberto.pasinelli@vogelwarte.ch; Phone: +41 41 462 97 58

APPENDIX S1 – Supplemental Tables

Table S1. Number of surveys and territories as well as densities in territories per hectare (terr/ha) of *Phylloscopus bonelli* and *P. sibilatrix* per subsite per year in the Swiss Jura Mountains (2017-2019). Includes both marked and unmarked individuals.

|  |  |  | *P. bonelli* | |  | *P. sibilatrix* | |
| --- | --- | --- | --- | --- | --- | --- | --- |
| Subsite | Year | Number of surveys | Number of territories | Density (terr/ha) |  | Number of territories | Density (terr/ha) |
| EW_L | 2017 | 3 | 22 | 0.39 |  | 3 | 0.05 |
| EW_L | 2018 | 10 | 42 | 0.75 |  | 1 | 0.02 |
| EW_L | 2019 | 8 | 30 | 0.54 |  | 2 | 0.04 |
| EW_RT | 2017 | 3 | 40 | 0.48 |  | 8 | 0.10 |
| EW_RT | 2018 | 9 | 69 | 0.83 |  | 9 | 0.11 |
| EW_RT | 2019 | 10 | 47 | 0.57 |  | 2 | 0.02 |
| HB | 2018 | 10 | 13 | 0.22 |  | 3 | 0.05 |
| KL_A | 2017 | 3 | 15 | 0.50 |  | 1 | 0.03 |
| KL_A | 2018 | 4 | 24 | 0.81 |  | 4 | 0.13 |
| KL_A | 2019 | 10 | 30 | 1.01 |  | 2 | 0.07 |

Table S1, continued.

|  |  |  | *P. bonelli* | |  | *P. sibilatrix* | |
| --- | --- | --- | --- | --- | --- | --- | --- |
| Subsite | Year | Number of surveys | Number of territories | Density (terr/ha) |  | Number of territories | Density (terr/ha) |
| KL_D | 2017 | 3 | 5 | 0.06 |  | 0 | 0.00 |
| KL_D | 2019 | 6 | 10 | 0.11 |  | 0 | 0.00 |
| KL_Dh | 2019 | 8 | 17 | 0.61 |  | 5 | 0.18 |
| KL_S | 2017 | 3 | 13 | 0.34 |  | 0 | 0.00 |
| KL_S | 2018 | 4 | 16 | 0.42 |  | 2 | 0.05 |
| KL_S | 2019 | 9 | 22 | 0.58 |  | 0 | 0.00 |
| LB | 2017 | 3 | 9 | 0.18 |  | 1 | 0.02 |
| LB | 2018 | 9 | 10 | 0.20 |  | 1 | 0.02 |
| LW | 2018 | 10 | 37 | 0.57 |  | 5 | 0.08 |
| LW | 2019 | 10 | 44 | 0.68 |  | 3 | 0.05 |
| MS | 2017 | 3 | 31 | 0.23 |  | 1 | 0.01 |
| MS | 2018 | 4 | 23 | 0.17 |  | 2 | 0.01 |
| MS | 2019 | 9 | 31 | 0.23 |  | 4 | 0.03 |
| SP | 2019 | 3 | 66 | 0.46 |  | 8 | 0.06 |

Table S2. Biometric data (mean values ± standard error) for male (M) and female (F) *Phylloscopus bonelli* and *P. sibilatrix* breeding in the Swiss Jura Mountains*.*

|  | Species | | | | | | |
| --- | --- | --- | --- | --- | --- | --- | --- |
|  | *P. bonelli* | | |  | *P. sibilatrix* | | |
|  | M |  | F |  | M |  | F |
|  | n=52 |  | n=28 |  | n=84 |  | n=49 |
| Metric |  |  |  |  |  |  |  |
| Wing length | 64.78±0.23 |  | 60.00±0.28 |  | 75.50±0.18 |  | 71.53±0.29 |
| Tail length | 49.41±0.21 |  | 46.21±0.26 |  | 49.04±0.20 |  | 47.04±0.31 |
| Tarsus length | 18.95±0.07 |  | 18.72±0.11 |  | 18.06±0.06 |  | 18.14±0.08 |
| Bill length | 7.77±0.04 |  | 7.03±0.05 |  | 7.30±0.02 |  | 6.89±0.03 |
| Bill width | 2.57±0.02 |  | 2.58±0.03 |  | 2.61±0.02 |  | 2.72±0.03 |
| Bill depth | 2.46±0.24 |  | 2.46±0.10 |  | 2.58±0.20 |  | 2.62±0.06 |

Table S3. Variables included as fixed effects in Bayesian generalized linear mixed models predicting aggressive responses song playbacks in male *Phylloscopus sibilatrix* and *P. bonelli*.

| Variable name | Description |
| --- | --- |
| Playback type | Three-level categorical variable indicating type of playback stimulus: Typical heterospecific song, Typical conspecific song, or *P. sibilatrix* Mixed Song |
| Species | Two-level categorical variable indicating if focal bird was “*sibilatrix*” or “*bonelli*” |
| Time period | Binary variable indicating if a male was tested in the first or second half of the three week-long period when testing took place (1: Apr 30 – May 12; 2: May 12 – May 23) |
| Test order | Three-level categorical variable indicating the order in which a given playback treatment was presented (1st, 2nd, or 3rd) |
| Female nearby* | Binary variable indicating if a fertile female was nearby (either a focal male’s own mate or the mate of a neighboring male). A female was considered fertile if she was in the process of building a nest or had a finished nest with no eggs in it yet (see Luepold et al. 2024). |
| Neighbor interference | Binary variable indicating if a neighboring male came in and responded aggressively to the playback by either direct attack, flyovers or singing (“1”) or not (“0”) |

*Included because males may be focused on female and, therefore, not respond to song playback

Table S4. Degree of evidence for the null hypothesis (H_0_) and alternative hypothesis (H_1_) associated with a given range of Bayes Factor (BF) values (based on Jeffreys 1961).

| BF Value | Degree of Evidence for H_0_ or H_1_ |
| --- | --- |
| >100 | Decisive evidence for H_1_ |
| 30-100 | Very strong evidence for H_1_ |
| 10-30 | Strong evidence for H_1_ |
| 3-10 | Moderate evidence for H_1_ |
| 1-3 | Weak evidence for H_1_ |
| 0.33-1 | Weak evidence for H_0_ |
| 0.10-0.33 | Moderate evidence for H_0_ |
| 0.03-0.10 | Strong evidence for H_0_ |
| 0.01-0.03 | Very strong evidence for H_0_ |
| <0.01 | Decisive evidence for H_0_ |

APPENDIX S2

*Dynamic time-warping analysis of acoustic similarity (Fig. 3 in main text)*

The analysis included a total of 15 *P. sibilatrix* males (8 mixed singers, 7 individuals with species-typical song) and 9 *P. bonelli* males. Prior to analysis, we visually scanned spectrograms of 26 locally recorded *P. bonelli* males and classified syllabe types using a catalogue of *P. bonelli* syllable types created by S. Carlotti. We also visually compared spectrograms of mixed songs to *P. bonelli* syllable types to determine which type they most closely resembled. Most mixed singers (64%, n=11) sang a syllable that was visually similar to a *P. bonelli* “type B” syllable. Two mixed singers sang a “type W” syllable, one sang a “type A” syllable and one sang a “type D” syllable. In the analysis, we included *P. sibilatrix* mixed songs containing type B or type W syllables, *P. bonelli* songs containing type B or W syllables, and *P. sibilatrix* songs containing species-typical songs.

For each male included in the analysis, we selected three songs of good acoustic quality (clear signal, no background noise) for spectrogram measurements. For the 6 individuals that had two syllable types included in the analysis (4 *P. sibilatrix* mixed singers, 2 *P. bonelli*), we selected three songs of each type. From each song, we selected three syllables of good signal quality for measurement. Thus, we analyzed 9 syllables per male per type.

In Luscinia, spectrograms were visualized using a Gaussian window function with the following (default) settings: maximum frequency 15 KHz, 5 ms frame length, 221 spectrograph points, 80% spectrograph overlap, 50 dB dynamic range, 50% dereverberation, 50 ms dereverberation range and a 2.5 KHz High Pass threshold. Using a brush size of 2, we traced individual elements (elements are defined as an unbroken stretch of signal, Lachlan 2007). For syllables with multiple distinct elements (e.g., type W), elements were grouped into syllables for analysis.

We used the dynamic time-warping (DTW) algorithm in Luscinia to compare syllables of species-typical *P. sibilatrix* songs, *P. sibilatrix* mixed songs and *P. bonelli* songs. This algorithm compares each point in a signal to each point in the other signals in a sample, generating a matrix. From this matrix, the distance (i.e., similarity) between two signals is calculated by finding the most efficient path (i.e., lowest cumulative distance) through the matrix. We based our choice of DTW settings in Luscinia on Bliard *et al.* (2021) and Lachlan (2007). We weighted acoustic features as follows: time (5), mean frequency (1), normalized mean frequency (1) and mean frequency change (2). All other acoustic features were weighted by their standard deviation. Additional settings included: compression factor of 0.001, minimum element length of 10 ms, Time SD weighting of 1, Non-time SD weighting of 0, ArcTan transform weight of 0.02, syllable repetition weighting of 0, maximum warp of 25%, cost for stitching syllables of 0, and cost of alignment error of 0.2. Syllables were compared by stitching elements.

From the dissimilarity matrix, the program generated two principal components via nonmetric multidimensional scaling. We exported the PC values for each syllable to R, and then calculated the mean values per song per male. We then averaged the values per song to obtain average values per male per syllable type and plotted these average values.

APPENDIX S3 – Supplemental Figures


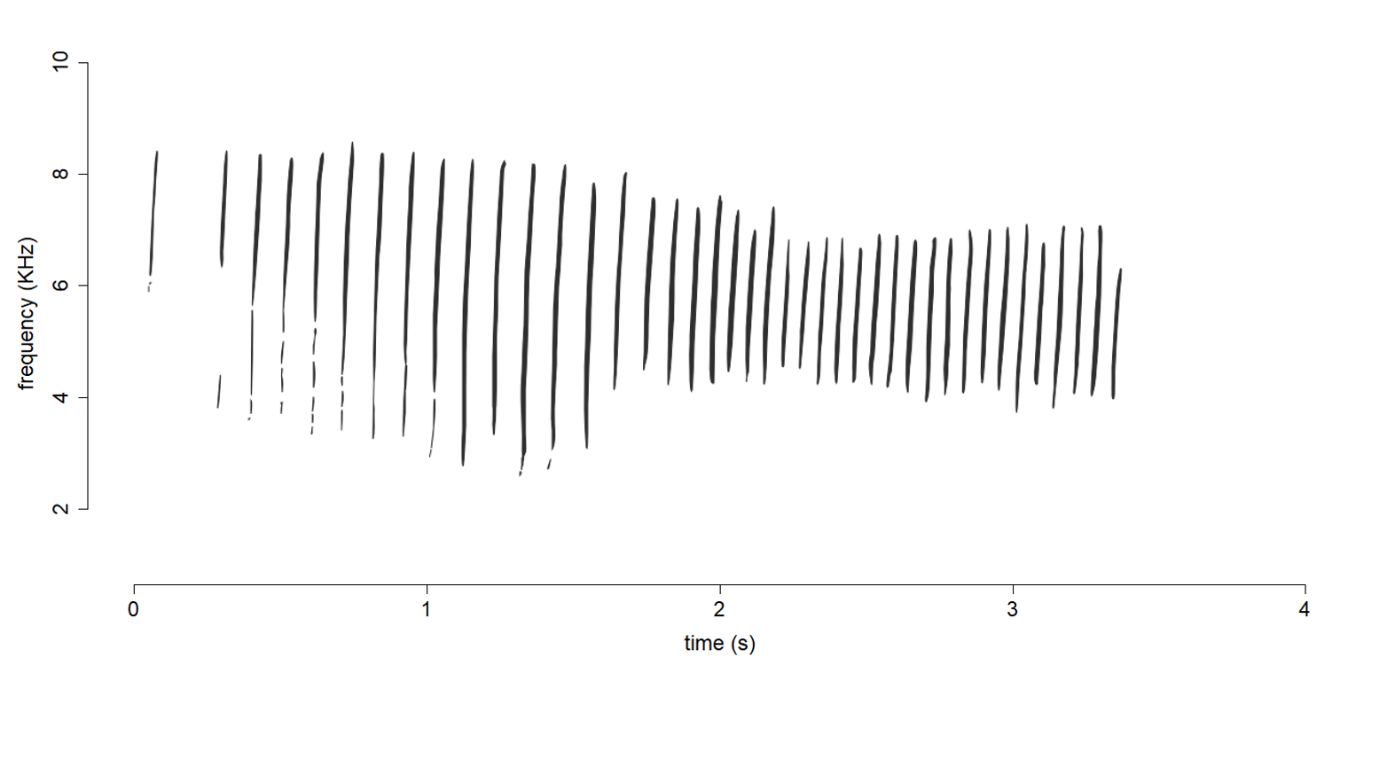


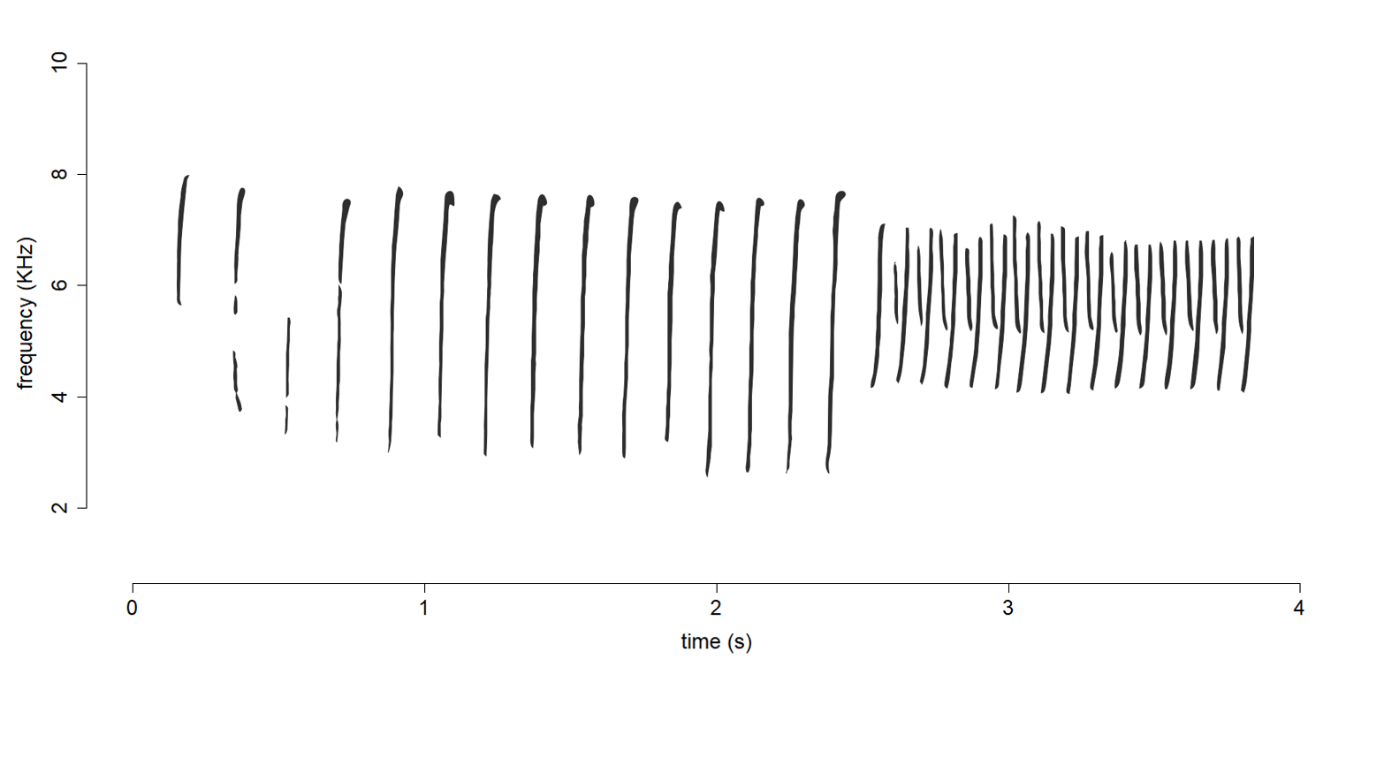


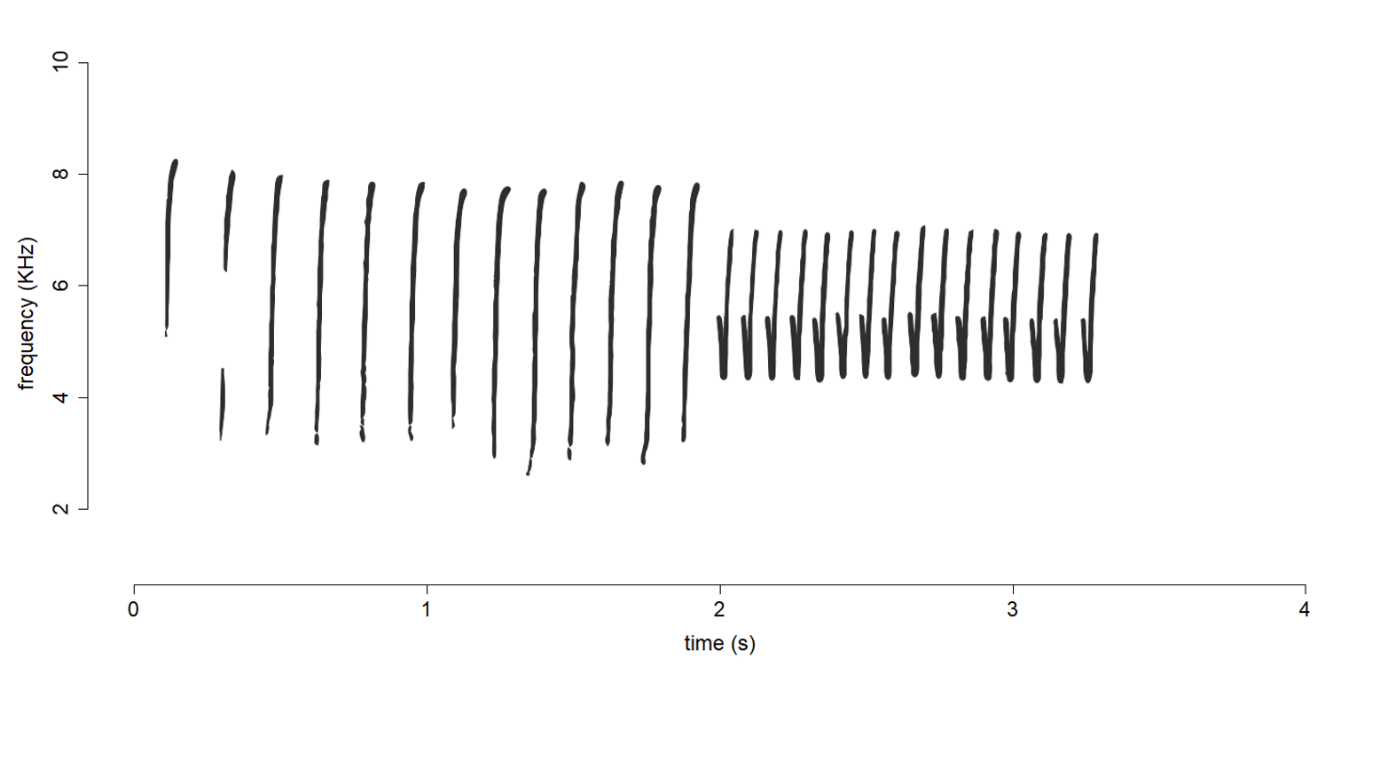


Fig. S1. Spectrograms of species-typical *P. sibilatrix* song (top), *P. sibilatrix* mixed song with *P. bonelli* syllables “W” (middle) and “B” (bottom) in part 2 of the song.


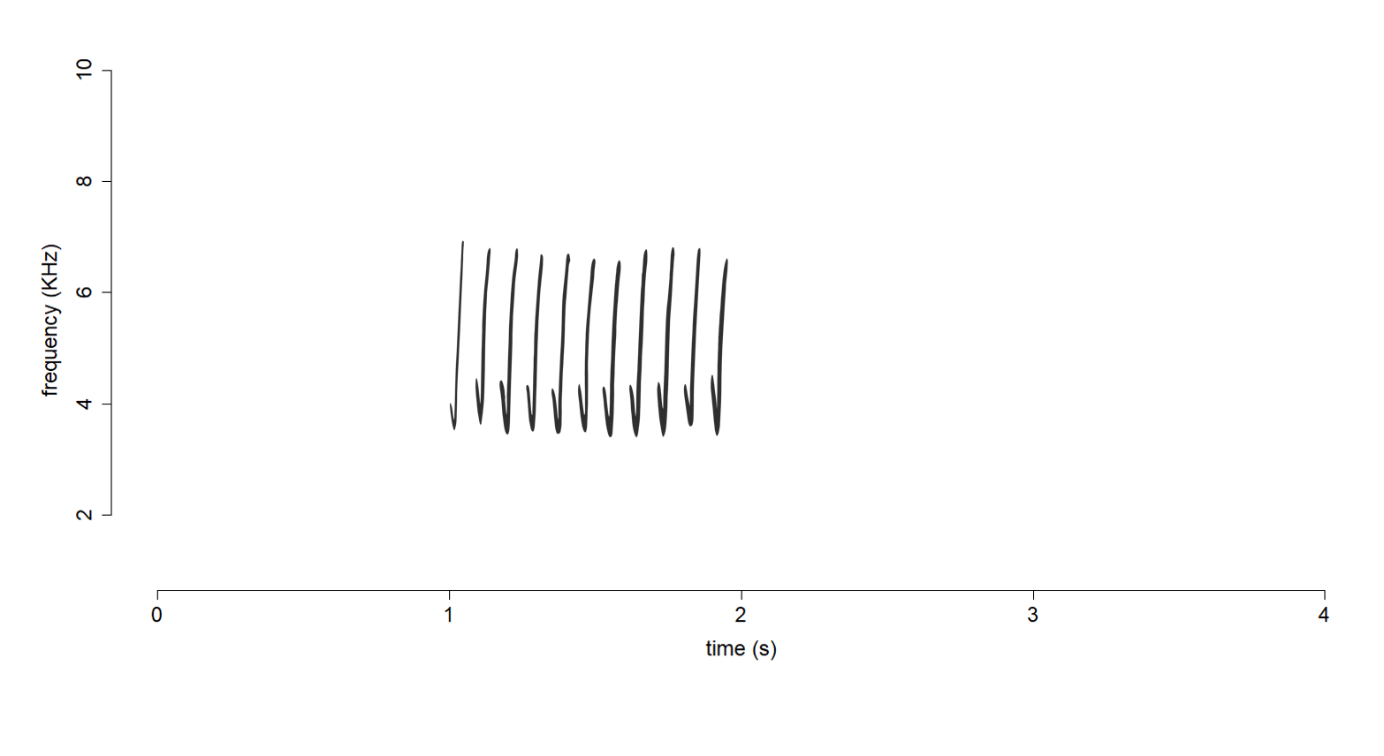

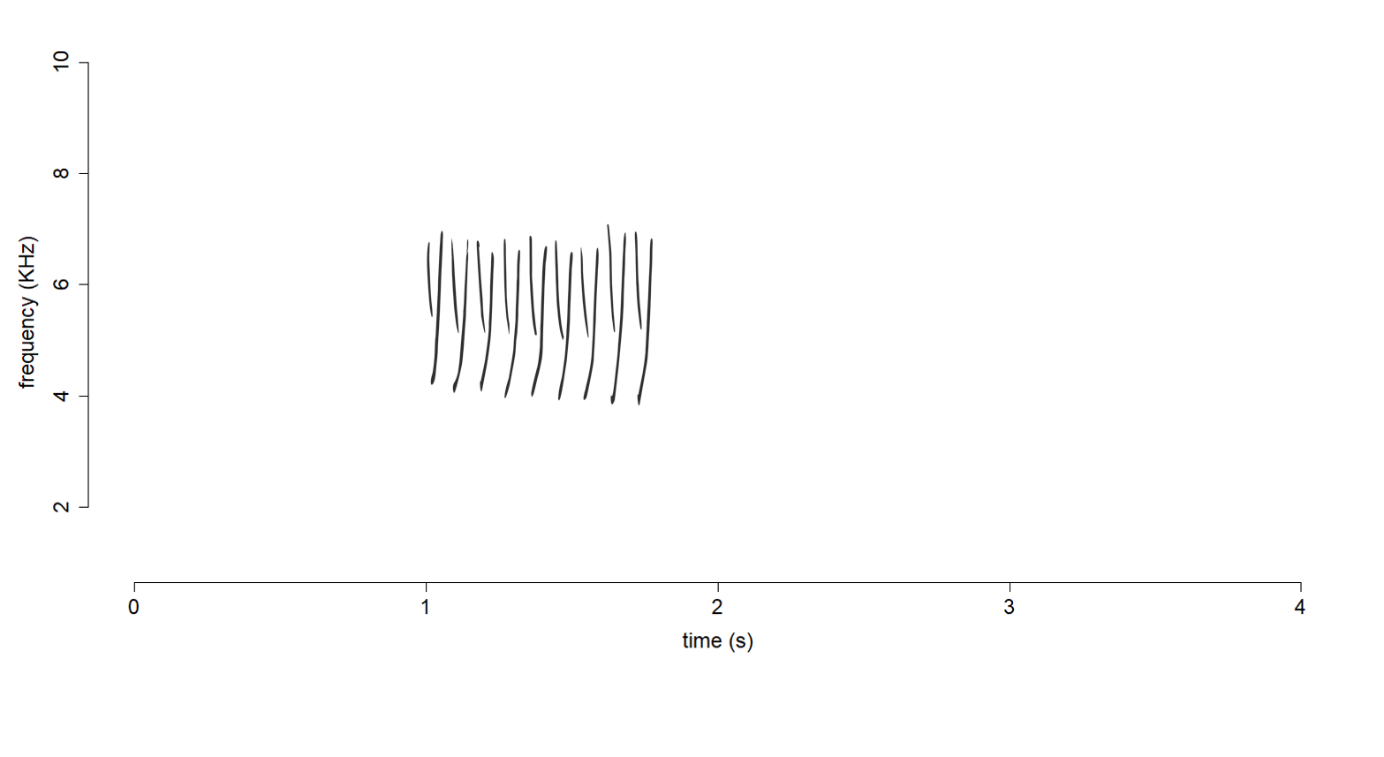


Fig. S2. Spectrograms of species-typical *P. bonelli* song with syllables “W” (top) and “B” (bottom).


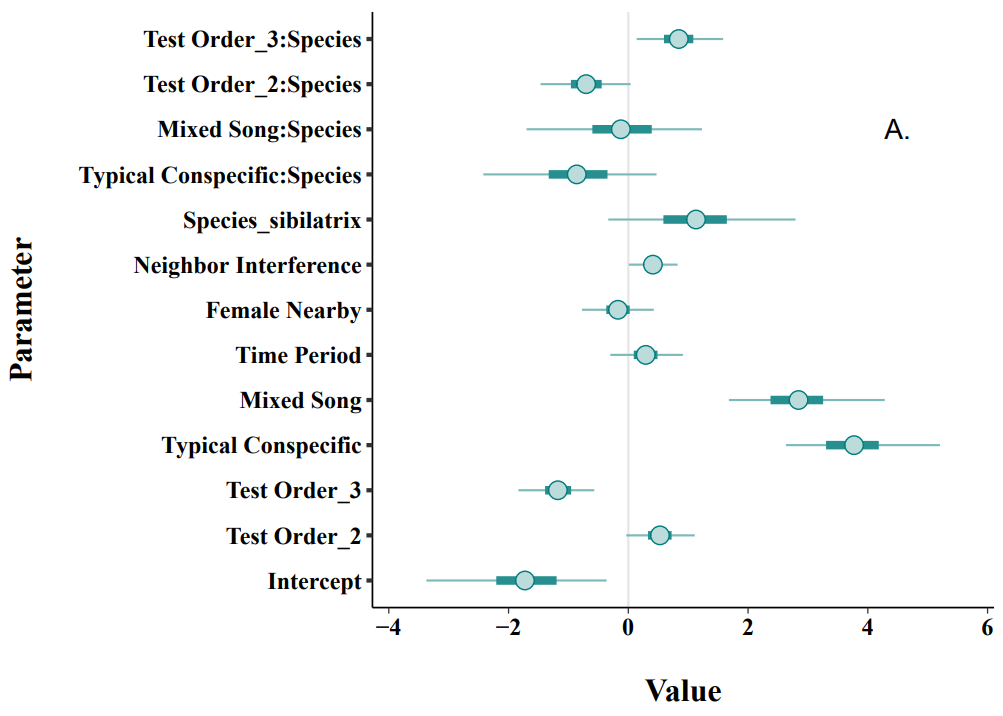


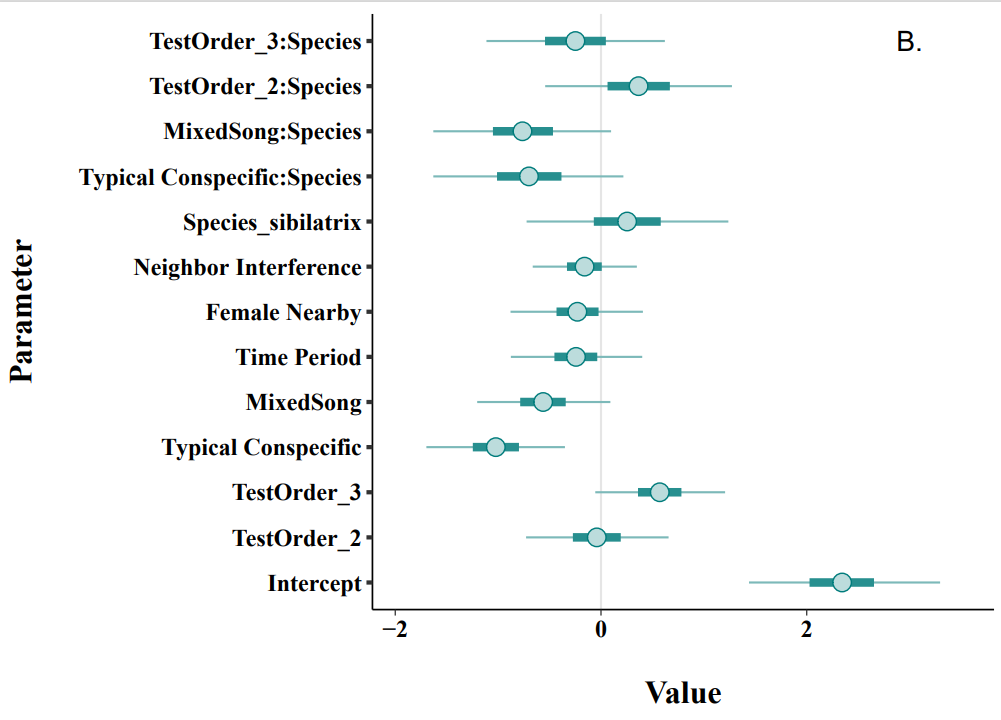


Figure S3. Mean of the posterior distribution and quantile-based credible intervals for each parameter in the model predicting (A) number of flyovers and (B) minimum distance to speaker. Points represent the mean, thick lines indicate the 50% intervals and thin lines indicate the 95% credible interval. *P. bonelli* is the reference level for the binary variable Species (such that “Species_sibilatrix” is the response of *P. sibilatrix* relative to *P. bonelli*).


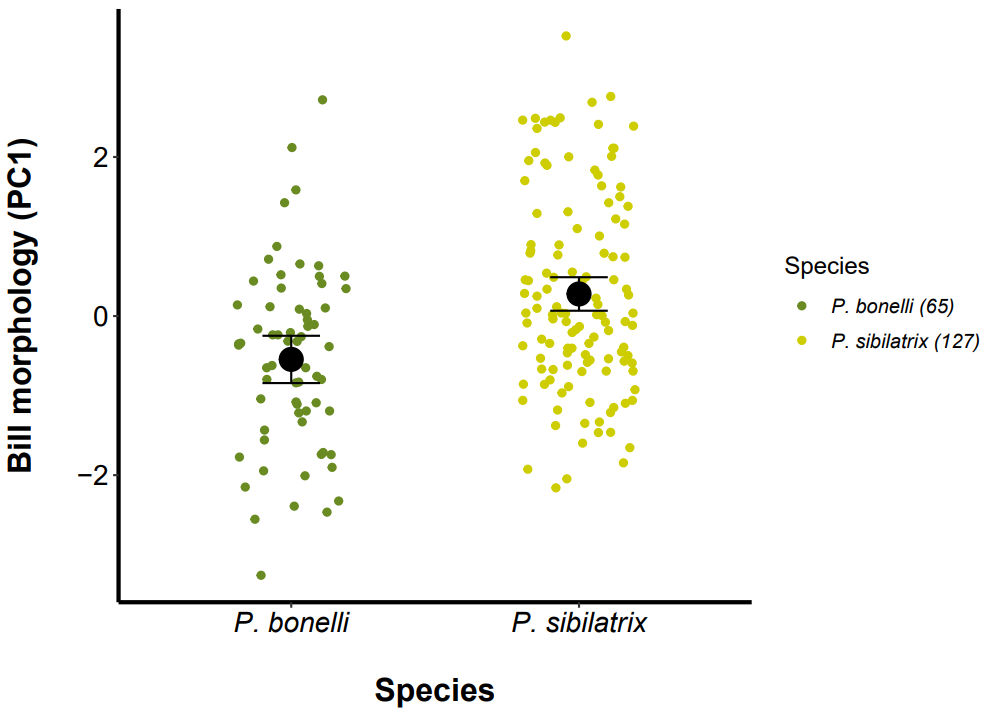


Figure S4. PC1 (principal component 1 based on bill length, width and depth) in relation to

species. Large circles indicate predictions (mean of the predictive posterior

distribution) of a linear model relating PC1 to species, error bars represent uncertainty intervals (i.e., range of values with 2.5 to 97.5% probability) and dots are raw data points. Data for both species includes bill measurements from males and females. Raw data points are jittered to reduce point overlap.

Variable loadings for PC1 are as follows: bill length=0.32; bill width=0.64; bill depth=0.70.

REFERENCES

Bliard L, Qvarnström A, Wheatcroft D. 2021. The role of introductory alarm calls for song discrimination in *Ficedula* flycatchers. Anim Behav. 177:241 – 252.

Jeffreys H. 1961. Theory of probability. 3rd. Oxford, U. K.: Oxford University Press.

Lachlan RF. 2007. Luscinia: a bioacoustics analysis computer program. v2.16.10.29.01. github.com/rflachlan/Luscinia.
